# First Incidence of Peste des Petits Ruminants Virus in *Cervidae* Family from State Zoo of Assam, India

**DOI:** 10.3390/v16121829

**Published:** 2024-11-25

**Authors:** Nagendra Nath Barman, Arpita Bharali, Durlav Prasad Bora, Biswajit Dutta, Mousumi Bora, Sophia M. Gogoi, Panchami Sharma, Sankar Sarma, Parikshit Kakati, Tejas Mariswamy, Ankita Choudhury, Lukumoni Buragohain

**Affiliations:** 1 College of Veterinary Science, Assam Agricultural University, Guwahati 781022, Assam, India; arpita.bharali@gmail.com (A.B.); durlav.bora@aau.ac.in (D.P.B.); drbiswajitkvk@gmail.com (B.D.); sophiagogoi@gmail.com (S.M.G.); choudhuryankita6@gmail.com (A.C.); 2 College of Veterinary Science, Guru Angad Dev Veterinary and Animal Sciences University, Ludhiana 141004, Punjab, India; mousumeebora11@gmail.com; 3 Assam State Zoo-cum-Botanical Garden, Guwahati 781005, Assam, India; panchami23@gmail.com (P.S.); mail2sankar11@rediffmail.com (S.S.); tejasm24@gmail.com (T.M.); 4 World Wildlife Fund (WWF-India), Guwahati 781029, Assam, India; parik67@gmail.com

**Keywords:** Assam State Zoo, cervids, lineage IV, peste des petits ruminants virus, spillover

## Abstract

The present study aimed to investigate the episodes of per-acute mortality due to peste des petits ruminants (PPR) that resulted in the death of 30 animals of different species of cervids, namely, barking deer, four-horned antelope, hog deer, thamin, and mouse deer in the State Zoo of Assam, a northeastern state of India. The affected animals showed no to limited clinical signs. However, the necropsy and histopathological findings were highly suggestive of PPR virus (PPRV) infection observed in domestic small ruminants. Representative tissue samples were screened for the presence of PPRV along with blue tongue virus (BTV) and epizootic hemorrhagic disease virus (EHDV) using RT-PCR or RT-qPCR and were found to be positive for PPRV. Considering the sudden outbreak of PPR in captive cervids, we sought to determine the role of domestic goats as the potential spillover host. To verify that, archived tissue samples of domestic goats collected during PPRV outbreaks in nearby localities and slaughtered goats used as meat for Carnivorous animals in the State Zoo were also screened and found to be positive for PPRV in RT-PCR. Phylogenetic analysis based on the Nucleocapsid (N) protein gene of PPRV from infected cervids, domestic goats, and goat meat revealed the virus to be of Lineage IV origin. Our findings provide evidence of probable spillover of PPRV from domestic goats to captive endangered cervids and circulation of Lineage IV PPRV strains among the small-ruminant population of this region.

## 1. Introduction

Peste des petits ruminants (PPR) is a highly contagious and economically devastating disease of small ruminants [1]. PPR is included on the World Organization for Animal Health (WOAH) notifiable disease list because of its rapid transmission and high morbidity and mortality in affected animals [2]. About 80% (1.7 billion) of the global small ruminant population is at risk of PPR [3]. PPR was first reported in the Ivory Coast, West Africa, in 1942. Since its first report, it has spread to more than 70 countries in Africa, the Middle East, and South Asia, indicating its immense transboundary potential [4]. PPR is caused by peste des petits ruminants virus (PPRV), a morbillivirus within the family *Paramyxoviridae* which shares an antigenic relationship with other members of the genus rinderpest and measles virus [5]. PPRV exists as a single serotype and has been differentiated into four distinct lineages (I–IV) based on the characterization of the Fusion (F) and or Nucleocapsid (N) gene [6,7]. Lineages I–III were considered of African and Middle Eastern origin, whereas Lineage IV was found to be prevalent in Asian countries [3]. Considering the severe economic impacts of PPR in countries whose economy relies on small-ruminant farming and production, the World Organization for Animal Health (WOAH) and the Food and Agricultural Organization of United States (FAO) have launched PPR-Global Eradication Program (PPR-GEP) in 2015 with a mission to eradicate PPR by the year 2030 [4].

PPR is currently endemic to most of Saharan and sub-Saharan Africa, Turkey, the Middle East, and the Indian subcontinents [8]. Similar to other morbillivirus infections, PPRV needs close contact between infected and susceptible animals to spread [9]. The virus can be transmitted directly through aerosols and indirectly through contaminated fomites, feed, and water. The incubation period can range from 3–10 days [10]. Secretions and excretions of sick animals are the sources of infection, which can also occur during the incubation period. The clinical disease is manifested by sudden onset of fever (up to 41 °C), oculonasal discharges, stomatitis, pneumonia with foul, offensive breath, and inflammation of the gastrointestinal tract leading to severe diarrhea followed by death or recovery [11,12].

Initially, only domestic sheep and goats were presumed to be the primary hosts of PPRV [9]. However, over the past few decades, PPR has been reported in multiple wildlife ruminant species and has been confirmed by the detection of the genome, viral antigens, isolation of the virus, and serology [13,14]. Growing evidence of PPRV infecting a diverse host range and its expansion to the free-ranging wildlife are major concerns to the Peste des Petits Ruminants Global Eradication Program (PPR-GEP) [15]. In the present study, we investigated outbreaks of PPR in five different species of the *Cervidae* family in the state zoo of Assam, a northeastern state of India. The current investigation has revealed that PPRV infection in captive zoo cervids is a result of potential spillover events from PPRV-infected domestic goats and their meat used as feed for carnivorous animals in the state zoo. The present study also indicates that risk assessment and targeted surveillance of PPRV in domestic small ruminants and meat and meat products would help to minimize the loss due to PPR and control the disease in both captive and wild ruminants.

## 2. Materials and Methods

### 2.1. Study Area and Outbreak Investigation

**Figure 1:**
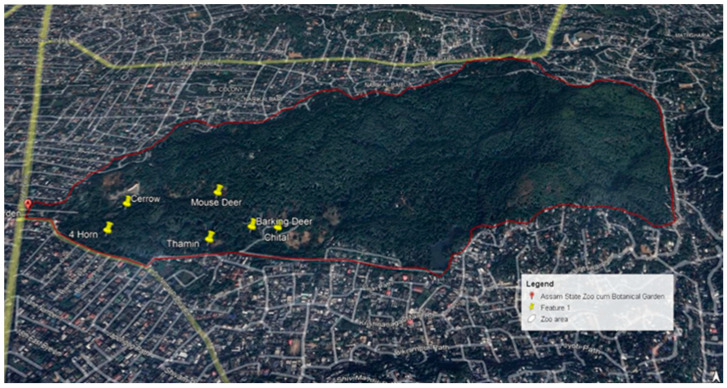
Map of Assam State Zoo cum Botanical Garden with location of dead animals in different species of cervids.

The Assam state zoo cum Botanical Garden (Land area 175 hectares), located at latitude 26°10′58.836″ N and longitude 91°46′31.548″ E in the Guwahati area of Kamrup Metropolitan district, has experienced a sudden disease outbreak among the cervid population comprising of seven different species, namely, barking deer, four-horned antelope, hog deer, thamin, mouse deer, serow and chital. The population of cervids is accommodated in the westernmost region of the zoo within a well-defined enclosed space bounded by a fence (Figure 1). The barking deer population is reared as free-rangers, i.e., they cannot be handled, tested, or effectively medicated by the zoo staff. They are free-living within the enclosed space and can roam freely within the area. The other species of cervids were kept in enclosures as captive inmates within the enclosed space that separates the cervid population from the rest of the captive animals of the zoo. Among the seven species of cervids, five species, namely barking deer, four-horned antelope, hog deer, thamin, and mouse deer, were infected in the present outbreak.

### 2.2. Necropsy and Histopathology

The carcasses of PPRV suspected cervids were collected for necropsy investigation. Necropsy was performed by trained veterinarians in pathology and carried out in the post-mortem facility located inside the zoo premises. The tissue samples of the lungs, liver, heart, brain, intestine, kidney, spleen, and lymph nodes were collected in duplicate for histopathology and molecular analysis. For histopathological studies, tissue specimens were collected from the lung, liver, heart, and brain, portions from the small intestine, kidney, and lymph nodes of cervids carcasses of different species. Tissue samples were trimmed into a thin cube piece of about 2 mm on the side and fixed in 10% *v*/*v* buffered formalin. Formalin-fixed paraffin-embedded (FFPE) 4–5 μm sections were prepared using a rotary microtome (Thermo Scientific Shandom^TM^ Finesse^TM^ 325 manual microtome, Cheshire, UK) and placed on polylysine-coated slides for routine histological staining (hematoxylin–eosin; H&E) [16]. The slides were examined under a microscope (Labovision, Haryana, India), and images were captured (Tucsen, Fuzhou, Fujian, China) and analyzed (TCapture v5.1.1).

### 2.3. Molecular Analysis

Considering the sudden disease outbreak in different species of deer, we sought to determine the role of domestic goats as potential spillover hosts of the infectious pathogen. To track the possible spillover, additional samples (tissue, *n* = 4) from slaughtered goats, which were used as meat for the Carnivorous animals in the state zoo, were also collected to test for the presence of PPRV. To confirm the outbreak of PPR, samples collected from affected cervids and slaughtered goats were screened for the presence of PPRV along with bluetongue virus (BTV) and enzootic hemorrhagic disease virus (EHDV) for differential diagnosis.

To determine the probable spillover, additional samples from two PPRV-infected domestic goats, available in the repository of Advanced Animal Disease Diagnosis and Management Consortium (ADMaC) Laboratory, College of Veterinary Science, Assam, India, were taken into consideration for molecular analysis. These samples were collected from a previous outbreak that occurred in January 2021 in a nearby district to Guwahati, namely, Mangaldoi in the Darrang district of Assam.

#### 2.3.1. RNA Extraction and cDNA Synthesis

Representative tissue samples (spleen and lymph nodes) from 30 animals belonging to different species of cervids, along with four tissue samples of slaughtered goats and two repository tissue samples, were processed for RNA extraction using QIAamp^®^ Viral RNA Mini Kit (Qiagen, Hilden, Germany). The extracted RNA was reverse transcribed into cDNA by using the RevertAid First Strand cDNA Synthesis Kit (Thermo Scientific, Waltham, MA, USA). The reaction mixture consists of total RNA (6 μL), random hexamer primer (1 μL), nuclease-free water (5 μL), 5X Reaction Buffer (4 μL), RiboLock RNAase inhibitor (1U) (1 μL), 10 mM dNTP mix (2 μL) and RevertAid M-MuLV RT (200 U/μL) (1 μL). The contents were mixed properly and spun briefly and then placed in the thermal cycler (Veriti™, Applied Biosystem, Carlsbad, CA, USA) for incubation on the following conditions: 25 °C for 5 min, 42 °C for 60 min, 70 °C for 5 min followed by the final hold at 4 °C. The synthesized cDNA was stored at −20 °C until further use.

#### 2.3.2. Reverse Transcription Polymerase Chain Reaction (RT-PCR) and RT-Quantitative PCR (RT-qPCR)

For the detection of PPRV by RT-PCR, the N gene was targeted, which can detect all four lineages of PPRV by using the primer sets, NP3 (5′-GTCTCGGAAATCGCCTCACAGACT-3′) and NP4 (5′-CCTCCTCCTGGTCCTCCAGAATCT-3′) as describe in WOAH Terrestrial Manual, 2019 [17]. The PCR reaction consisted of 25 µL DreamTaq PCR Master Mix (2X) (Thermo Scientific, USA), 2 µL of each NP3 and NP4 primer (10 µM), 5 µL of cDNA template, and the final volume was adjusted up to 50 µL with nuclease-free water. The PCR was run in a Thermal cycler (Veriti™, Applied Biosystem, USA) following the method described in the OIE Terrestrial Manual (2013) with minor alterations. Briefly, initial denaturation at 95 °C for 10 min, followed by 40 cycles of 94 °C for 30 s, 60 °C for 30 s, and 72 °C for 45 s, and a final extension of 72 °C for 10 min and hold at 4 °C. The amplified RT-PCR products were resolved in 1.5% agarose gel electrophoresis stained with ethidium bromide and finally observed under UV light in the Gel-Doc System (Bio-Rad, Hercules, CA, USA).

For the detection of BTV, RT-qPCR was performed by using the ViroReal^®^ Kit Bluetongue Virus (BTV) (Ingenetix, Wien, Austria) as per the manufacturer’s instruction. This kit targets the N3 segment of BTV. Similarly, for detection of EHDV by RT-qPCR, the Techne^®^ qPCR Kit for Epizootic Hemorrhagic Disease Virus targeting the NS1 segment of EHDV was used as per the instructions provided by the manufacturer.

#### 2.3.3. Sequencing and Phylogenetic Analysis of PPRV

The representative samples (one each from barking deer, four-horned antelope, hog deer, thamin and mouse deer, one from slaughtered goat meat, and two from PPRV-infected domestic goats) (*n* = 8) were used for sequencing. The PCR amplified N gene products of PPRV were subjected to agarose gel electrophoresis followed by gel purification of the amplified products using QIAquick^®^ gel extraction kit (Qiagen, Germany). The purified PCR products were subjected to bidirectional Sanger sequencing, which was outsourced (1st Base, Seri Kembangan, Selangor, Malaysia). The sequencing data were analyzed by using the BLASTn program and BioEdit Software v7.2.5. For phylogenetic analysis, the sequences were trimmed to 255 nt (that encodes a small variable region in the C-terminus of the N protein), which is considered one of the phylogenetic makers for PPRV [18]. The partial N gene sequences of PPRV belonging to different Lineages (I–IV) were retrieved from the NCBI GenBank database (https://www.ncbi.nlm.nih.gov/ accessed on 16 January 2024). The phylogenetic analysis was performed in MEGA 11 software [19] with 61 partial N gene sequences of PPRV, and out of 61 sequences, eight (8) sequences were generated in this study. Alignment of multiple sequences was carried out using the CLUSTALW program present in MEGA 11 software. All positions that contained alignment gaps and missing data were eliminated completely from the analysis. The neighbor-joining (NJ) method [20] was used for constructing the phylogenetic tree, and the substitution model used was Kimura 2-parameter with gamma distribution (K2+G) [21]. The reliability of the tree was evaluated by bootstrap [22] with 1000 replicates. The pairwise distance of partial N gene sequences of lineage IV was estimated in MEGA 11 software.

## 3. Results

### 3.1. Mortality in Various Species of Cervids

Seven different species of Cervidae in the state zoo of Assam were distributed in different locations within a defined enclosed space that accommodates the cervid population of the zoo. Out of seven species, five different species, namely barking deer, four-horned antelope, hog deer, thamin, and mouse deer, were affected by PPR. However, serow and chital remained unaffected throughout the period. The first mortality was reported on 28 February 2021 when three barking deer were found dead. Subsequently, mortality in different species of cervids was recorded, as shown in Table 1. The outbreak continued till 19 March 2021. The highest mortality was recorded in four-horned antelope (84.21%), followed by mouse deer (21.73%), hog deer (9.52%), barking deer (6.25%), and thamin (7.01%).

### 3.2. Clinical Manifestation of PPRV Infections in Affected Cervids

**Figure 2:**
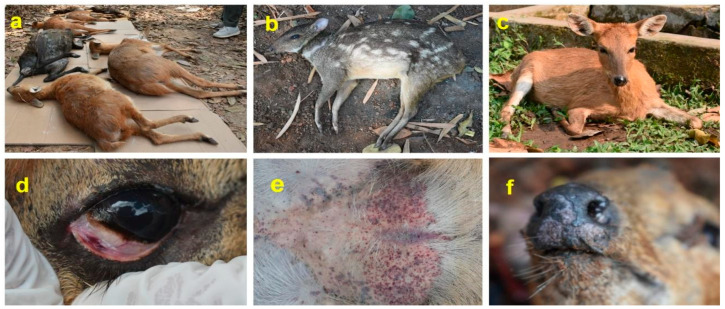
Clinical manifestation of peste des petits ruminants virus infections in affected cervids. (**a**) Sudden death of four-horned antelopes (**b**) A death mouse deer due to PPR (**c**) An ailing four-horned antelope (**d**) Hemorrhages in the palpebral conjunctiva of an ailing four-horned antelope (**e**) Hemorrhages in ventral region and (**f**) Sloughing of the external nasal epithelium.

Initially, two species from the affected cervids, i.e., barking deer and four-horned antelope, showed no visible symptoms of PPR, and episodes of sudden deaths were observed. However, lately, several other affected cervids have shown signs of ocular discharge, nasal secretions, buccal lesions, salivation, and diarrhea. In addition, two female horned antelope have aborted well-developed fetuses (Figure 2a–f).

### 3.3. Necropsy Findings

**Figure 3:**
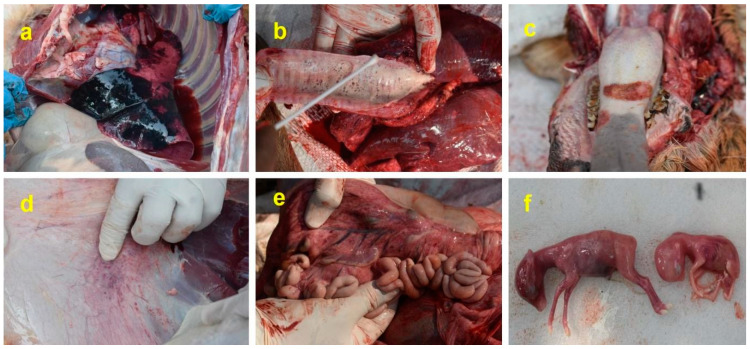
Necropsy lesions observed in peste des petits ruminants virus-infected cervids: (**a**) Hemorrhages and congestion of the lung parenchyma; (**b**) Frothy trachea with congested lungs; (**c**) Ulcerative lesions on the tongue; (**d**) Hemorrhages on the peritoneum; (**e**) Hemorrhages and congestion on the omentum and mesentery; and (**f**) Fetal death following PPRV infection in four-horned antelopes.

On necropsy analysis, extensive subcutaneous hemorrhages in the abdominal area and mucous membrane of the eyes were observed in the affected cervids. Congestion of lungs and frothy trachea were observed in all the affected species. Ulcerative lesions were observed on the tongue and hard palate. Hemorrhagic lesions were observed in the lungs, omentum, mesentery, endocardium, mesenteric lymph nodes, liver, and mucosal surface of the urinary bladder and intestines. Interestingly, two of the pregnant four-horned antelope ewes displayed vertical transmission of infection from the dam to the fetus. Extensive hemorrhages were observed in the aborted fetus apart from hemorrhagic lesions in multiple organs of both the dams, along with ulcers on the tongue (Figure 3a–f).

### 3.4. Histopathological Findings

**Figure 4:**
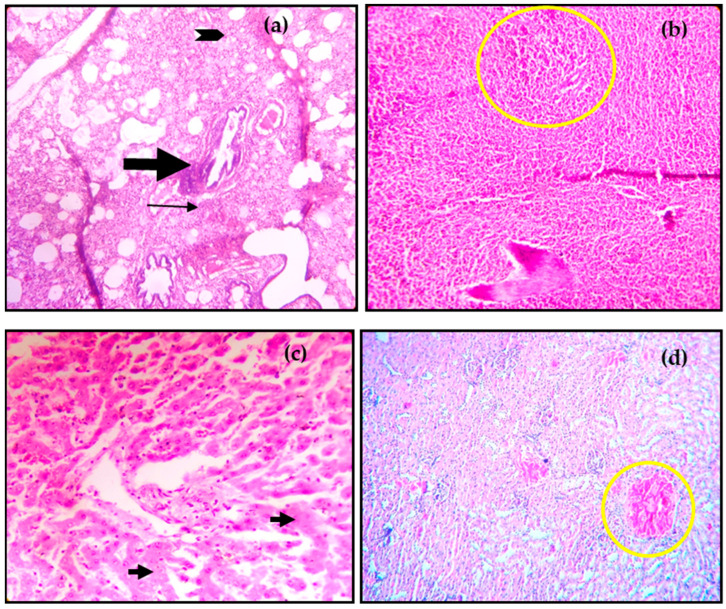
Histopathological changes in different tissues in peste des petits ruminants virus infected: (**a**) Presence of serous exudate in alveolar and bronchiolar spaces (arrowhead), hyperplasia of the bronchiolar epithelium (thick arrow) with thickening of interalveolar septa (thin arrow) due to infiltration of mononuclear cells in the lung (H&E, 10×); (**b**) Depletion of lymphocytes from the lymphoid follicle (yellow circle) of the spleen (H&E, 10×); (**c**) Presence of focal area of coagulative necrosis (arrows) in liver (H&E, 40×); and (**d**) Dilatation and marked congestion (yellow circle) of renal capillaries (H&E, 10×).

In histopathology, congestion and diffuse areas of hemorrhages throughout the lung parenchyma and bronchiolar hyperplasia were observed (Figure 4a). The presence of serous exudates in bronchiolar and alveolar spaces with numerous mononuclear infiltrations was evident. The alveolar septa were thickened with edema, hemorrhages, and infiltrating cells, indicating interstitial pneumonia. The lymphoid follicles showed a marked depletion of lymphocytes (Figure 4b). The liver showed marked congestion, vascular degeneration at different areas, focal areas of hepatic necrosis, disruption of hepatic cords, and formation of acini (Figure 4c). In the brain, focal areas of hemorrhages with mild demyelination and focal neuronophagia were recorded. Dilatation and congestion were observed in the kidneys (Figure 4d).

### 3.5. Molecular Analysis

In the case of PPRV-positive samples, PCR amplicons of 351 bp were observed. All the samples from four-horned antelope (*n* = 4), mouse deer (*n* = 1), hog deer (*n* = 2), barking deer (*n* = 1) and thamin (*n* = 2) showed positive amplification. Interestingly, one of the goat meat samples that was served to the other carnivorous species of the zoo was also found to be positive for PPRV in RT-PCR. Moreover, the domestic goat samples taken from the repository of ADMaC Laboratory were also reconfirmed to be PPRV positive in RT-PCR. However, all the tested samples were found to be negative for BTV and EHDV.

**Figure 5:**
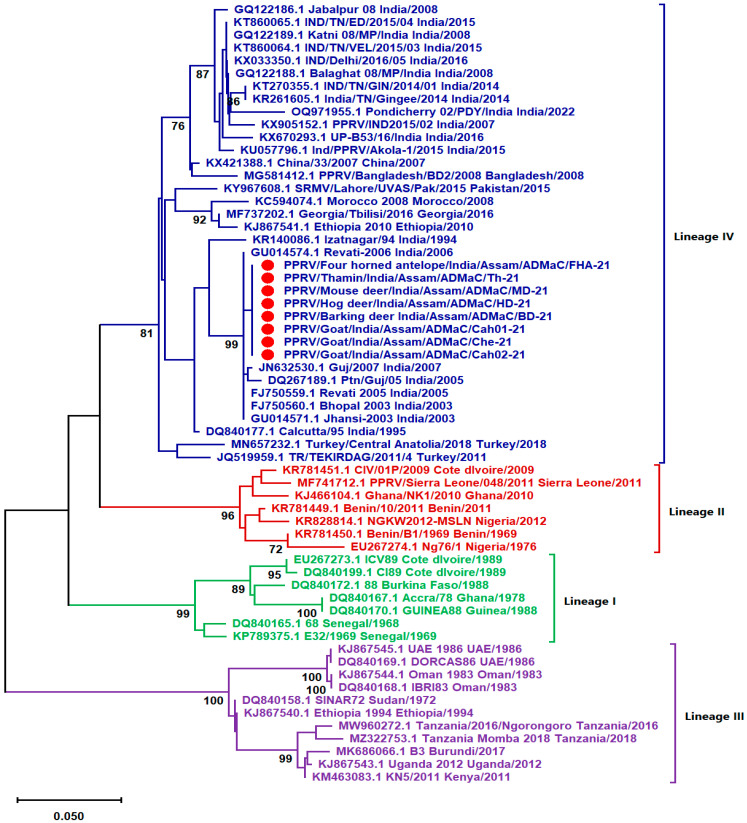
Phylogenetic analysis of PPRV strains performed in MEGA 11 based on deduced nucleotide sequences of the partial Nucleocapsid (N) gene. Clades with four different colors represent four lineages (I–IV) of PPRV, and the eight PPRV strains detected in this study formed a clade with the lineage IV (blue-colored clade), which is highlighted with a solid red circular marker.

Phylogenetic analysis performed with 61 partial N gene (255 nucleotides) sequences revealed that all the strains of PPRV i.e., PPRV/Four-horned antelope/India/Assam/ADMaC/FHA-21, PPRV/Hog deer/India/Assam/ADMaC//HD-21, PPRV/Thamin/India/Assam/ADMaC/Th-21, PPRV/Mouse deer/India/Assam/ADMaC/MD-21, and PPRV/Barking deer India/Assam/ADMaC/BD-21 detected in different species of cervids, namely, four-horned antelope, hog deer, thamin, mouse deer and barking deer, respectively belong to the lineage IV (Figure 5). The PPRV strain (PPRV/Goat/India/Assam/ADMaC/Che-21) detected in goat meat samples also belongs to lineage IV. Moreover, the strains PPRV/Goat/India/Assam/ADMaC/Cah01-21 and PPRV/Goat/India/Assam/ADMaC/Cah02-21 detected in domestic goats which were collected from an earlier outbreak also formed clade within lineage IV PPRV strains.

The pairwise distance analysis based on partial N gene sequences revealed that the divergence among lineage IV of PPRV strains ranges from 0.00–0.105 (Supplementary Table S1). All the eight strains that were detected in this study from different species were 100% identical to each other.

## 4. Discussion

PPR is one of the economically devastating diseases of sheep and goats, and it poses an immense challenge for successful small ruminant production. After the successful eradication of rinderpest, the WOAH and FAO have targeted PPR as the next animal disease to be eradicated from the world by the year 2030 [4]. The availability of potent live attenuated vaccines, reliable diagnostics, single serotype of PPRV, and extensive scientific knowledge make it an ideal candidate for eradication [23]. However, the challenge in the eradication process remains with the continual spread of the disease in newer territories and its detection in wildlife and unusual hosts over the past decades. The PPR-related deaths reported in much-endangered wildlife, which include goitered gazelle, bharal, ibex, wild goat, and recently Mongolian antelope, and probably many others, further highlights the scourge it causes to biodiversity [24,25].

In the present study, epidemiological, pathomorphological, and molecular findings support the diagnosis of PPRV infections and spillover events from domestic to free-ranging and captive cervids. All the infected cervids, namely, barking deer, four-horned antelope, hog deer, thamin, and mouse deer, were confirmed to be susceptible and manifested clinical signs similar to typical PPRV infections in domestic sheep and goats that include ocular to nasal discharge, erosion, and necrosis of oral mucosa, the sudden death of apparently healthy animals and abortions. The infected cervids were found to be capable of spreading PPRV infection within their population within a short period of time. The highest mortality (84.21%) was observed in captive four-horned antelope, suggesting high viral excretion loads over the period of the outbreaks. Previous reports of PPRV infection in different species of antelope in a semi-captive setting also showed a high level of susceptibility, as recorded in our present report [26]. The other infected species of cervids, such as barking deer in free-ranging settings and hog deer, thamin, and mouse deer in captive settings, respectively showed comparatively lower mortality rates, which, however, confirms the susceptibility of these species.

The study has demonstrated that there were initial episodes of sudden deaths in barking deer and four-horned antelope, presenting a fatal per-acute to acute infection, indicating possibilities of virus shedding in feces or discharges that lead to further virus transmission to several other species of cervids. Subsequently, the other species of cervids displayed typical PPR clinical presentation in small ruminants characterized by ocular and nasal discharges, oral lesions, salivation, and diarrhea. Similar clinical signs were observed in the previous literature where sudden onset and fatal PPRV infection were encountered in dromedary camels in Africa [27,28]. This indicates that sudden onset and high mortalities can be reported in herds that have not been exposed before to PPRV, irrespective of lineages.

PPRV infection in some of the reported instances has killed wildlife like Gazelles, Bharals, Sindh ibex, Ibex, Afghan markhor, Wild goat, and Mongolian antelope acutely within a short incubation period. Some infections and mortalities in wildlife, as reported in bharals [29] and more recently in Saiga antelope [30], may be a regular feature in the wildlife. The wildlife dying acutely either in protected settings or under free range might be of less importance in transmission cycles but poses a tragic risk of cataclysm to the wildlife, particularly for endangered species, and thus a significant challenge for biodiversity conservation. On the other hand, increased morbidity in wildlife may complicate the situation with active virus shedding, leading to active and continuous transmissions in the wild niche. Hence, the epidemiological dimension of PPR in relation to wildlife, especially regarding endangered species, requires immediate attention for the reasons of biodiversity conservation and PPRV emergence in the wild niche.

The gross pathology in free-ranging and captive cervids was highly indicative and consistent with typically reported PPRV lesions in domestic small ruminants. Extensive subcutaneous hemorrhage in the abdominal area and mucous membrane of the eyes, as observed in the infected cervids, was largely consistent with the necropsy findings in domestic goats [31]. The other notable necropsy findings, such as ulcerative lesions on the tongue, frothy trachea, hemorrhages and congestion of the lungs, hemorrhages and congestion in omentum and mesentery, and abortions, are more similar to those that have been observed in sheep and goats manifesting with PPR [31,32,33]. At histopathology, the presence of serous exudates in bronchiolar and alveolar spaces with mononuclear infiltrations was observed since PPRV localizes in the lung parenchyma and causes interstitial pneumonia [33,34,35]. Bronchiolar hyperplasia and depletion of lymphocytes from lymphoid organs are indicative of an active PPRV infection and affinity of the virus towards respiratory and lymphoid tissues [33,36]. A similar observation of lymphoid depletion was recorded in the Mongolian Saiga antelope and led to virulent expression of PPRV in infected animals [30].

The partial N gene sequences of PPRV from each species of infected cervids were identical to sequences obtained from domestic goats during outbreaks of PPR in nearby areas and districts surrounding the Guwahati area in 2021. The sequences from infected cervids were also found to be identical to partial N gene sequences of PPRV isolated from slaughtered goat meat samples used as feed for Carnivorous animals in the State Zoo. This indicates a spillover of PPRV from infected domesticated goats to the cervids. The sequences from the present study showed 100% identity with each other and were genetically similar to sequences of PPRV of Lineage IV origin previously reported from India, supporting the evidence of circulation of similar PRRV strain among the goat population of India. Comparative sequence analysis of the partial N gene sequences of PPRV from the infected animals revealed a close relationship among the sequences of Lineage IV origin from various regions of Europe and Africa, indicating the dominance of the Asian Lineage IV beyond its geographic distribution [18,37,38].

This spillover event could be a result of three probable activities: (i) handling of infected domestic goats, (ii) transport of contaminated meat, and (iii) feeding of cervids with contaminated feed. Even though the major route of PPRV transmission is through direct contact between infectious and susceptible animals, the disease majorly spreads through human activities such as livestock trade, animal movements for the purpose of breeding, social functions, selling and purchasing livestock without observing quarantine measures and ineffective livestock raising practices [39]. Goat farming in Assam is primarily a low-input-driven production system dominated by marginal farmers for the purpose of livelihood and revenue generation. Prior to the outbreak of PPR in the state zoo, the surrounding districts of Guwahati region of Assam have been reporting outbreaks of PPR in the domestic goat population. This has probably surged the sale of goats incubating the disease with or without clinical manifestation, which is usually a practice the marginal farmers do in order to avoid economic losses associated with a disease. The infected goats might have mixed up with a healthy herd of goats during the trading of livestock in markets and slaughter points eventually drives highly contagious PPRV to introduce into new geographic areas. Therefore, transmission of PPRV by zoo animal handlers while procuring and handling PPRV-contaminated goat meat in the feed preparation facility is extremely likely. The transportation of such contaminated meat samples to the state zoo to feed the carnivores along with ruminant feed in the common vehicle without appropriate biosecurity measures could be another factor in the spillover event. Further, spillover of PPRV to the cervid population could also be possible through procuring contaminated meat prepared in a common space by the same animal handlers preparing feed for wild ruminants. PPRV is thermosensitive, and its half-life is estimated to be only 3 h at 37 °C [40]. Even though there is very little information on the survivability of PPRV in infected carcasses, transmission of the virus might be possible at low ambient temperatures since the virus is capable of surviving for several months in frozen and salted meat [41]. Therefore, the chances of PPRV survival in infected goat meat samples during the period of PPR outbreaks from January to March 2021 are likely since the ambient temperature is low during winters in Assam. However, in the present study, a partial gene (N gene) of PPRV was sequenced, which is not sufficient to support the data on transmission likelihood. The possible transmission events could have been tracked, and it could have been proved whether whole genome sequencing of the isolated strains had been performed.

The present study of the spillover event of PPRV from domestic goats to the cervid population of Assam State Zoo indicates a lapse in the biosecurity measures by animal handlers, which might include hand hygiene, cleaning spills of blood or body substances, appropriate management and disposal of waste material, safe use and disinfection of equipment for feed preparation, managing accidental exposure to infected animals and care with the movement of feed items from one location to another. Animal handlers should be aware of situations where appropriate or enhanced disease risk assessment and management is required. Wildlife infected with PPRV should be considered for higher biosecurity risk, and enhanced measures should be put in place. Therefore, zoo authorities are encouraged to assess their biosecurity risks and to develop and maintain an optimum level of biosecurity for their operations.

The growing list of reports describing PPR affecting wildlife represents an important component in the epidemiology of the disease [13]. Increasing evidence of interspecies transmission of PPRV to bovines [42,43], swine [44], and camelids [45,46] are major concerns to PPR-GEP. It is, therefore, important to take the necessary steps to prevent the loss of biodiversity, which is integral to sustainable development. The recent die-offs implicated to PPR with >900 deaths in Saiga antelopes in Mongolia are quite alarming and indicate possible challenges for PPR eradication. In India, reports of PPRV in wildlife have been previously reported from Indian Buffaloes [47], dromedary camels [48], Asiatic lions [49], Indian cattle [50], and Chowsingha [51]. The evolutionary emergence of PPRV strains in wildlife can have a high impact on small ruminant populations throughout the endemic regions where frequent mixing of wildlife with domestic small ruminants occurs. Such situations warrant more attention for the surveillance of PPR emergence in wild caprines like bharal, ibex, wild goat, etc. The mortalities in free-ranging and captive cervids in our present study are a wake-up call for all stakeholders to strengthen approaches in wildlife disease surveillance, disease dynamics, and risk assessment in livestock-wildlife interfaces. The prevalence of PPR in natural hosts and infections in wildlife hosts give scope for more understanding and critical thinking when national, regional, and global disease control and eradication plans are in the process of implementation.

## Figures and Tables

**Table 1:** Mortality rate of various species of Cervidae due to PPR outbreaks in the State Zoo, Assam, India.

Duration of Outbreak	Species	Scientific Name	Habitat	Total Population	Mortality			
					Sub-Adult	Adult	Total	Percent (%)
February 2021	Barking deer	*Muntiacus muntjak*	Free-ranging	48	1	2	3	6.25
March 2021	Four-horned Antelope	*Tetracerus quadricornis*	Captive	19	3	13	16	84.21
March 2021	Hog Deer	*Axis porcinus*	Captive	21	-	2	2	9.52
March 2021	Thamin	*Rucervus eldii*	Captive	57	-	4	4	7.01
March 2021	Mouse deer	*Moschiola indica*	Captive	23	-	5	5	21.73

Sub-adult: 6 months–2 years; Adult: 2–4 years.

## Data Availability

Data are contained within the article and the Supplementary Materials.

## Supplementary Materials

The following supporting information can be downloaded at https://www.mdpi.com/article/10.3390/v16121829/s1. Table S1: The pairwise distance of Lineage IV strains of PPRV based on the N gene.
