# Supplementary material for: First Incidence of Peste des Petits Ruminants Virus in Cervidae Family from State Zoo of Assam, India
Source: Viruses. 2024 Nov 25;16(12):1829. doi: 10.3390/v16121829 (PMC11680224; doi:10.3390/v16121829)
Supplement: Supplementary file 1 [file viruses-16-01829-s001.zip › viruses-3263069-supplementary.pdf]

Supplementary Table S1. The pairwise distance of Lineage IV strains of PPRV based on N gene.

PPRV Strains/Isolates	1	2	3	4	5	6	7	8	9	10	11	12	13	14	15	16	17	18	19	20	21	22	23	24	25	26	27	28	29	30	31	32	33	34	35	36
GQ122186.1_Jabalpur_08_India/2008		0.006	0.006	0.006	0.006	0.006	0.008	0.008	0.010	0.007	0.012	0.017	0.017	0.015	0.017	0.019	0.019	0.019	0.019	0.020	0.020	0.020	0.020	0.020	0.020	0.020	0.020	0.016	0.018	0.020	0.016	0.017	0.009	0.012	0.020	0.008
KT860065.1_IND/TN/ED/2015/04_India/2015	0.008		0.000	0.000	0.000	0.000	0.006	0.006	0.008	0.007	0.010	0.017	0.017	0.015	0.017	0.019	0.019	0.019	0.019	0.020	0.020	0.020	0.020	0.020	0.020	0.020	0.020	0.016	0.020	0.020	0.017	0.019	0.009	0.013	0.021	0.006
GQ122189.1_Katni_08/MP/India_India/2008	0.008	0.000		0.000	0.000	0.000	0.006	0.006	0.008	0.007	0.010	0.017	0.017	0.015	0.017	0.019	0.019	0.019	0.019	0.020	0.020	0.020	0.020	0.020	0.020	0.020	0.020	0.016	0.020	0.020	0.017	0.019	0.009	0.013	0.021	0.006
KT860064.1_IND/TN/VEL/2015/03_India/2015	0.008	0.000	0.000		0.000	0.000	0.006	0.006	0.008	0.007	0.010	0.017	0.017	0.015	0.017	0.019	0.019	0.019	0.019	0.020	0.020	0.020	0.020	0.020	0.020	0.020	0.020	0.016	0.020	0.020	0.017	0.019	0.009	0.013	0.021	0.006
KX033350.1_IND/Delhi/2016/05_India/2016	0.008	0.000	0.000	0.000		0.000	0.006	0.006	0.008	0.007	0.010	0.017	0.017	0.015	0.017	0.019	0.019	0.019	0.019	0.020	0.020	0.020	0.020	0.020	0.020	0.020	0.020	0.016	0.020	0.020	0.017	0.019	0.009	0.013	0.021	0.006
GQ122188.1_Balaghat_08/MP/India_India/2008	0.008	0.000	0.000	0.000	0.000		0.006	0.006	0.008	0.007	0.010	0.017	0.017	0.015	0.017	0.019	0.019	0.019	0.019	0.020	0.020	0.020	0.020	0.020	0.020	0.020	0.020	0.016	0.020	0.020	0.017	0.019	0.009	0.013	0.021	0.006
KT270355.1_IND/TN/GIN/2014/01_India/2014	0.016	0.008	0.008	0.008	0.008	0.008		0.000	0.010	0.009	0.012	0.019	0.019	0.016	0.019	0.021	0.021	0.021	0.021	0.022	0.022	0.022	0.022	0.022	0.022	0.022	0.022	0.017	0.021	0.022	0.018	0.020	0.011	0.015	0.023	0.008
KR261605.1_India/TN/Gingee/2014_India/2014	0.016	0.008	0.008	0.008	0.008	0.008	0.000		0.010	0.009	0.012	0.019	0.019	0.016	0.019	0.021	0.021	0.021	0.021	0.022	0.022	0.022	0.022	0.022	0.022	0.022	0.022	0.017	0.021	0.022	0.018	0.020	0.011	0.015	0.023	0.008
KX670293.1_UP-B53/16/India_India/2016	0.024	0.016	0.016	0.016	0.016	0.016	0.024	0.024		0.011	0.014	0.020	0.020	0.018	0.020	0.020	0.020	0.020	0.020	0.021	0.021	0.021	0.021	0.021	0.021	0.021	0.021	0.017	0.021	0.021	0.018	0.020	0.012	0.015	0.022	0.010
KX905152.1_PPRV/IND2015/02_India/2007	0.012	0.012	0.012	0.012	0.012	0.012	0.020	0.020	0.029		0.013	0.020	0.019	0.018	0.019	0.021	0.021	0.021	0.021	0.022	0.022	0.022	0.022	0.022	0.022	0.022	0.022	0.018	0.019	0.022	0.018	0.020	0.011	0.015	0.023	0.009
OQ971955.1_Pondicherry_02/PDY/India_India/2022	0.033	0.024	0.024	0.024	0.024	0.024	0.033	0.033	0.042	0.037		0.021	0.021	0.019	0.021	0.023	0.023	0.023	0.023	0.024	0.024	0.024	0.024	0.024	0.024	0.024	0.024	0.020	0.024	0.023	0.021	0.023	0.014	0.017	0.024	0.012
KY967608.1_SRMV/Lahore/UVAS/Pak/2015_Pakistan/2015	0.061	0.061	0.061	0.061	0.061	0.061	0.070	0.070	0.080	0.075	0.089		0.019	0.013	0.018	0.018	0.018	0.018	0.018	0.019	0.019	0.019	0.019	0.019	0.019	0.019	0.019	0.015	0.018	0.019	0.014	0.014	0.015	0.016	0.020	0.019
MN657232.1_Turkey/Central_Anatolia/2018_Turkey/2018	0.061	0.061	0.061	0.061	0.061	0.061	0.070	0.070	0.080	0.075	0.084	0.062		0.013	0.016	0.020	0.020	0.020	0.020	0.021	0.021	0.021	0.021	0.021	0.021	0.021	0.021	0.017	0.021	0.021	0.019	0.021	0.015	0.019	0.022	0.019
JQ519959.1_TR/TEKIRDAG/2011/4_Turkey/2011	0.056	0.056	0.056	0.056	0.056	0.056	0.060	0.060	0.074	0.070	0.084	0.038	0.038		0.015	0.019	0.019	0.019	0.019	0.019	0.019	0.019	0.019	0.019	0.019	0.019	0.019	0.015	0.019	0.019	0.016	0.018	0.013	0.015	0.020	0.017
KR140086.1_Izatnagar/94_India/1994	0.061	0.061	0.061	0.061	0.061	0.061	0.070	0.070	0.080	0.075	0.089	0.062	0.052	0.047		0.013	0.013	0.013	0.013	0.014	0.014	0.014	0.014	0.014	0.014	0.014	0.014	0.013	0.021	0.014	0.018	0.020	0.016	0.018	0.015	0.017
GU014574.1_Revati-2006_India/2006	0.070	0.070	0.070	0.070	0.070	0.070	0.080	0.080	0.080	0.085	0.100	0.062	0.072	0.066	0.034		0.000	0.000	0.000	0.004	0.004	0.004	0.004	0.004	0.004	0.004	0.004	0.011	0.021	0.004	0.018	0.020	0.018	0.021	0.006	0.019
FJ750559.1_Revati_2005_India/2005	0.070	0.070	0.070	0.070	0.070	0.070	0.080	0.080	0.080	0.085	0.100	0.062	0.072	0.066	0.034	0.000		0.000	0.000	0.004	0.004	0.004	0.004	0.004	0.004	0.004	0.004	0.011	0.021	0.004	0.018	0.020	0.018	0.021	0.006	0.019
FJ750560.1_Bhopal_2003_India/2003	0.070	0.070	0.070	0.070	0.070	0.070	0.080	0.080	0.080	0.085	0.100	0.062	0.072	0.066	0.034	0.000	0.000		0.000	0.004	0.004	0.004	0.004	0.004	0.004	0.004	0.004	0.011	0.021	0.004	0.018	0.020	0.018	0.021	0.006	0.019
GU014571.1_Jhansi-2003_India/2003	0.070	0.070	0.070	0.070	0.070	0.070	0.080	0.080	0.080	0.085	0.100	0.062	0.072	0.066	0.034	0.000	0.000	0.000		0.004	0.004	0.004	0.004	0.004	0.004	0.004	0.004	0.011	0.021	0.004	0.018	0.020	0.018	0.021	0.006	0.019
PPRV/Four_horned_antelope/India/Assam/ADMaC/FHA-21	0.076	0.076	0.076	0.076	0.076	0.076	0.085	0.085	0.085	0.091	0.105	0.067	0.077	0.071	0.038	0.004	0.004	0.004	0.004		0.000	0.000	0.000	0.000	0.000	0.000	0.000	0.012	0.022	0.006	0.019	0.021	0.018	0.022	0.007	0.020
PPRV/Thamin/India/Assam/ADMaC/Th-21	0.076	0.076	0.076	0.076	0.076	0.076	0.085	0.085	0.085	0.091	0.105	0.067	0.077	0.071	0.038	0.004	0.004	0.004	0.004	0.000		0.000	0.000	0.000	0.000	0.000	0.000	0.012	0.022	0.006	0.019	0.021	0.018	0.022	0.007	0.020
PPRV/Mouse_deer/India/Assam/ADMaC/MD-21	0.076	0.076	0.076	0.076	0.076	0.076	0.085	0.085	0.085	0.091	0.105	0.067	0.077	0.071	0.038	0.004	0.004	0.004	0.004	0.000	0.000		0.000	0.000	0.000	0.000	0.000	0.012	0.022	0.006	0.019	0.021	0.018	0.022	0.007	0.020
PPRV/Hog_deer/India/Assam/ADMaC/HD-21	0.076	0.076	0.076	0.076	0.076	0.076	0.085	0.085	0.085	0.091	0.105	0.067	0.077	0.071	0.038	0.004	0.004	0.004	0.004	0.000	0.000	0.000		0.000	0.000	0.000	0.000	0.012	0.022	0.006	0.019	0.021	0.018	0.022	0.007	0.020
PPRV/Barking_deer_India/Assam/ADMaC/BD-21	0.076	0.076	0.076	0.076	0.076	0.076	0.085	0.085	0.085	0.091	0.105	0.067	0.077	0.071	0.038	0.004	0.004	0.004	0.004	0.000	0.000	0.000	0.000		0.000	0.000	0.000	0.012	0.022	0.006	0.019	0.021	0.018	0.022	0.007	0.020
PPRV/Goat/India/Assam/ADMaC/Cah01-21	0.076	0.076	0.076	0.076	0.076	0.076	0.085	0.085	0.085	0.091	0.105	0.067	0.077	0.071	0.038	0.004	0.004	0.004	0.004	0.000	0.000	0.000	0.000	0.000		0.000	0.000	0.012	0.022	0.006	0.019	0.021	0.018	0.022	0.007	0.020
PPRV/Goat/India/Assam/ADMaC/Che-21	0.076	0.076	0.076	0.076	0.076	0.076	0.085	0.085	0.085	0.091	0.105	0.067	0.077	0.071	0.038	0.004	0.004	0.004	0.004	0.000	0.000	0.000	0.000	0.000	0.000		0.000	0.012	0.022	0.006	0.019	0.021	0.018	0.022	0.007	0.020
PPRV/Goat/India/Assam/ADMaC/Cah02-21	0.076	0.076	0.076	0.076	0.076	0.076	0.085	0.085	0.085	0.091	0.105	0.067	0.077	0.071	0.038	0.004	0.004	0.004	0.004	0.000	0.000	0.000	0.000	0.000	0.000	0.000		0.012	0.022	0.006	0.019	0.021	0.018	0.022	0.007	0.020
DQ840177.1_Calcutta/95_India/1995	0.051	0.051	0.051	0.051	0.051	0.051	0.060	0.060	0.060	0.065	0.079	0.043	0.052	0.047	0.034	0.025	0.025	0.025	0.025	0.029	0.029	0.029	0.029	0.029	0.029	0.029	0.029		0.017	0.012	0.014	0.016	0.014	0.016	0.013	0.015
KC594074.1_Morocco_2008_Morocco/2008	0.065	0.075	0.075	0.075	0.075	0.075	0.085	0.085	0.084	0.075	0.105	0.057	0.077	0.071	0.077	0.077	0.077	0.077	0.077	0.082	0.082	0.082	0.082	0.082	0.082	0.082	0.082	0.057		0.022	0.010	0.012	0.016	0.021	0.022	0.022
JN632530.1_Guj/2007_India/2007	0.075	0.075	0.075	0.075	0.075	0.075	0.085	0.085	0.084	0.090	0.105	0.066	0.077	0.071	0.038	0.004	0.004	0.004	0.004	0.008	0.008	0.008	0.008	0.008	0.008	0.008	0.008	0.029	0.081		0.019	0.021	0.018	0.021	0.006	0.020
MF737202.1_Georgia/Tbilisi/2016_Georgia/2016	0.051	0.060	0.060	0.060	0.060	0.060	0.070	0.070	0.069	0.065	0.089	0.042	0.061	0.056	0.061	0.061	0.061	0.061	0.061	0.066	0.066	0.066	0.066	0.066	0.066	0.066	0.066	0.042	0.020	0.066		0.006	0.016	0.018	0.020	0.019
KJ867541.1_Ethiopia_2010_Ethiopia/2010	0.060	0.070	0.070	0.070	0.070	0.070	0.079	0.079	0.079	0.074	0.099	0.042	0.071	0.066	0.071	0.071	0.071	0.071	0.071	0.076	0.076	0.076	0.076	0.076	0.076	0.076	0.076	0.051	0.029	0.076	0.008		0.017	0.020	0.022	0.021
KX421388.1_China/33/2007_China/2007	0.020	0.020	0.020	0.020	0.020	0.020	0.029	0.029	0.037	0.033	0.046	0.047	0.047	0.042	0.047	0.057	0.057	0.057	0.057	0.061	0.061	0.061	0.061	0.061	0.061	0.061	0.061	0.038	0.051	0.061	0.047	0.056		0.010	0.019	0.011
MG581412.1_PPRV/Bangladesh/BD2/2008_Bangladesh/2008	0.037	0.037	0.037	0.037	0.037	0.037	0.046	0.046	0.051	0.051	0.064	0.056	0.066	0.051	0.066	0.076	0.076	0.076	0.076	0.081	0.081	0.081	0.081	0.081	0.081	0.081	0.081	0.056	0.081	0.081	0.066	0.075	0.025		0.022	0.014
DQ267189.1_Ptn/Guj/05_India/2005	0.081	0.081	0.081	0.081	0.081	0.081	0.091	0.091	0.090	0.096	0.111	0.072	0.083	0.077	0.043	0.008	0.008	0.008	0.008	0.012	0.012	0.012	0.012	0.012	0.012	0.012	0.012	0.034	0.087	0.008	0.071	0.081	0.066	0.087		0.021
KU057796.1_Ind/PPRV/Akola-1/2015_India/2015	0.016	0.008	0.008	0.008	0.008	0.008	0.016	0.016	0.024	0.020	0.033	0.070	0.070	0.065	0.061	0.070	0.070	0.070	0.070	0.076	0.076	0.076	0.076	0.076	0.076	0.076	0.076	0.051	0.085	0.075	0.070	0.080	0.029	0.046	0.081	

NB: Values above the diagonal represent the standard error, and values below the diagonal represent the p-distance values obtained in MEGA 11 with 1000 bootstrap replicates.
